# Thyroid Dysfunction in Children with Chronic Kidney Disease: A Systematic Review and Meta-Analysis

**DOI:** 10.3390/medsci14060607

**Published:** 2026-09-27

**Authors:** Georgia Sotiriou, Vasiliki Karava, John Dotis

**Affiliations:** 1 First Department of Pediatrics, Aristotle University of Thessaloniki, Hippokration Hospital, 54642 Thessaloniki, Greece; gmsoti@gmail.com; 2 First Department of Pediatrics, “Aghia Sophia” Children’s Hospital, National and Kapodistrian University of Athens, 11527 Athens, Greece; vasilikikarava@hotmail.fr; 3 Third Department of Pediatrics, Aristotle University of Thessaloniki, Hippokration Hospital, 54642 Thessaloniki, Greece

**Keywords:** chronic kidney disease, children, subclinical hypothyroidism, thyroid function, prevalence, meta-analysis

## Abstract

Background/Objectives: Thyroid abnormalities have been reported in children with chronic kidney disease (CKD), but the broad term “thyroid dysfunction” encompasses clinically distinct phenotypes. This study aimed to estimate the prevalence of subclinical hypothyroidism (SCH) and characterize other thyroid abnormalities separately. Methods: PubMed/MEDLINE, Scopus, and Google Scholar were systematically searched according to PRISMA 2020. Eligible studies included patients aged 0–18 years with CKD stages 1–5, including kidney replacement therapy. The primary meta-analysis included peer-reviewed studies with an explicit or author-classified SCH definition and recoverable numerator and denominator data. Random-effects models used restricted maximum likelihood estimation with modified Hartung–Knapp inference. Results: Eleven studies involving 694 children were included. Seven peer-reviewed studies comprising 403 participants and 50 SCH cases contributed to the primary meta-analysis. The pooled SCH prevalence was 12.8% (95% CI, 9.2–17.6%), with a 95% prediction interval of 9.1–17.9% and no detected heterogeneity (I^2^ = 0%). A binomial-normal generalized linear mixed model yielded a similar estimate of 12.4% (95% CI, 9.3–15.9%). Including one preprint increased prevalence to 14.8% and introduced substantial heterogeneity (I^2^ = 65.9%). Overt hypothyroidism was reported sporadically, whereas low-T3/non-thyroidal-illness patterns were more frequent but unsuitable for quantitative synthesis. Conclusions: Approximately one in eight children in the included CKD cohorts was classified as having SCH according to study-specific definitions; persistence and clinical significance remain uncertain. Current evidence is insufficient to support routine CKD-specific levothyroxine therapy. Standardized longitudinal studies are needed to clarify persistence, clinical significance, and potential treatment benefits.

## 1. Introduction

Chronic kidney disease (CKD) in childhood is a lifelong disorder with consequences that extend well beyond impaired glomerular filtration. In addition to disturbances of fluid, electrolyte, mineral, and hematologic homeostasis, children with CKD are particularly vulnerable to endocrine complications that may affect linear growth, pubertal development, body composition, and long-term cardiovascular health. These complications assume particular importance in pediatrics because they occur during periods of rapid somatic and neurodevelopmental maturation. Contemporary CKD guidance therefore emphasizes the need to recognize and manage systemic complications across the entire disease course, including during conservative treatment and kidney replacement therapy [1,2].

The kidney and thyroid are linked through multiple, bidirectional physiological pathways. The kidneys contribute to iodine excretion, thyroid-hormone metabolism and clearance, and the peripheral conversion of thyroxine (T4) to the biologically more active triiodothyronine (T3). Conversely, thyroid hormones influence renal blood flow, tubular function, and glomerular filtration. Progressive kidney dysfunction may therefore alter thyroid biochemistry through reduced deiodination of T4 to T3, impaired iodine clearance, changes in hormone-binding proteins, chronic inflammation, malnutrition, metabolic acidosis, and disruption of hypothalamic–pituitary–thyroid signaling [3,4,5,6]. These mechanisms can generate several distinct biochemical patterns, including isolated low-T3 or low-T4 states, non-thyroidal illness, subclinical hypothyroidism (SCH), and overt hypothyroidism. Importantly, these phenotypes are not pathophysiologically or clinically interchangeable.

SCH is generally characterized by an elevated thyroid-stimulating hormone (TSH) concentration with a free thyroxine concentration within the reference range. In otherwise healthy children, mild SCH may be transient or persistent, and its clinical significance depends on the degree and duration of TSH elevation, underlying etiology, thyroid autoimmunity, symptoms, and associated conditions [7,8]. Interpretation is more complex in CKD because thyroid tests may be influenced by disease severity, intercurrent illness, protein loss, nutritional status, medications, iodine exposure, and dialysis-related factors. This distinction is particularly relevant when considering isolated low-T3 patterns, which may reflect altered peripheral metabolism rather than primary thyroid failure. Combining these abnormalities under the broad term “thyroid dysfunction” may therefore obscure clinically meaningful differences and produce prevalence estimates that depend heavily on how individual studies define and report thyroid phenotypes.

Pediatric evidence has accumulated gradually but remains fragmented. Early physiological work demonstrated abnormal thyroid-hormone profiles in children with chronic renal failure, including low-T3 and low-T4 patterns in clinically euthyroid patients [9]. Subsequent studies reported SCH or other hypothyroid phenotypes in children receiving dialysis and across non-dialysis CKD stages, but with considerable differences in participant selection, CKD severity, assay platforms, reference intervals, and diagnostic thresholds [10,11,12,13,14]. Some reports defined SCH using explicit TSH thresholds and preserved thyroid-hormone concentrations, whereas others used broader author-classified categories or did not provide sufficient laboratory detail to verify the phenotype. As a result, the pediatric literature has often treated SCH, overt hypothyroidism, and low-T3/non-thyroidal-illness patterns as components of a single outcome despite their biological differences. In contrast, recent meta-analytic evidence in dialysis populations is dominated by adults and includes very limited pediatric representation [15].

Accordingly, the burden of specifically defined SCH in children with CKD remains uncertain, and the extent to which published estimates are affected by phenotype definition and methodological variation has not been systematically quantified. A phenotype-specific synthesis is therefore needed to provide a clinically interpretable prevalence estimate while preserving other thyroid abnormalities as distinct outcomes. The present systematic review and meta-analysis aimed to determine the prevalence of SCH in children and adolescents with CKD using verified study-level definitions and recoverable numerators and denominators. Secondary objectives were to characterize other reported thyroid phenotypes, evaluate differences in diagnostic and laboratory methods across studies, and identify methodological limitations that should guide future pediatric CKD research.

## 2. Materials and Methods

### 2.1. Study Design, Registration, and Eligibility

This systematic review and meta-analysis was reported in accordance with the Preferred Reporting Items for Systematic Reviews and Meta-Analyses (PRISMA) 2020 statement [16]. Methods for prevalence outcomes were informed by JBI guidance [17,18]. The review was registered in PROSPERO (CRD420251240794); registration is reported for transparency, while the methods below describe the procedures applied to the verified evidence set and final analyses. A completed PRISMA 2020 checklist accompanies the manuscript as Supplementary File S1.

Observational studies were eligible if they included at least 10 children or adolescents aged 0–18 years with confirmed chronic kidney disease (CKD) stages 1–5 and reported thyroid-function data from which at least one relevant phenotype could be determined. Cross-sectional, cohort, and case–control designs were considered. Pediatric kidney replacement therapy (KRT) cohorts, including dialysis-only cohorts, were included within the CKD spectrum. Mixed-age studies were eligible only when pediatric data were reported separately. If healthy controls were enrolled, only participants with CKD contributed to prevalence denominators.

Peer-reviewed full reports were eligible for primary quantitative synthesis. A preprint with adequate methodological and numerical detail was included only in sensitivity analysis. Conference abstracts, reviews, editorials, case reports, case series with fewer than 10 participants, duplicate or overlapping cohorts, studies of acute kidney injury or renal conditions without established CKD, and reports without extractable pediatric thyroid data were excluded. For overlapping cohorts, the most complete report was retained. No language or geographic restrictions were applied.

Eligibility for the systematic review was distinguished from eligibility for specific quantitative synthesis. Thus, a report could remain included even when its phenotype definition or numerical reporting supported only descriptive analysis.

### 2.2. Information Sources, Search Strategy, and Study Selection

PubMed/MEDLINE, Scopus, and Google Scholar were searched on 31 March 2026, covering database inception through that date. For Google Scholar, the first 300 records ranked by relevance were screened. Reference lists of eligible reports and relevant reviews were examined, and targeted supplementary searching was completed on 1 August 2026. This supplementary search was an additional, non-prespecified check performed before submission and was not prompted by peer review. It involved targeted searches rather than a full repetition of the original Boolean strategies across all three sources. Controlled vocabulary, where available, and free-text terms were combined for three concepts: thyroid dysfunction or thyroid hormones, CKD or chronic renal insufficiency, and pediatric populations. Database-specific syntax was adapted without changing these concept blocks. Full search dates, complete line-by-line strategies, and source-specific record counts were retained in the review search log. The complete database-specific search strategies are provided in Supplementary Table S1.

The core Boolean logic was: (“thyroid dysfunction” OR hypothyroid* OR “subclinical hypothyroidism” OR “thyroid hormone*” OR TSH OR thyrotropin OR T3 OR triiodothyronine OR T4 OR thyroxine) AND (“chronic kidney disease” OR CKD OR “chronic renal insufficiency” OR “chronic renal failure” OR “kidney failure”) AND (child* OR adolescent* OR infant* OR pediatric* OR paediatric*).

After manual deduplication, two reviewers (G.S. and J.D.) independently screened titles and abstracts and assessed potentially eligible full texts. Disagreements were resolved by discussion and consensus, with third-reviewer (V.K.) adjudication when necessary. No additional eligible studies were identified through reference-list screening or targeted supplementary searching. Reports that remained unavailable after full-text searching were classified as not retrieved.

### 2.3. Outcomes, Data Extraction, and Verification

Full-text verification showed that the broad label “thyroid dysfunction” encompassed clinically distinct and inconsistently defined phenotypes. Outcome-specific synthesis rules were therefore applied before calculation of the definitive estimates. The primary outcome was subclinical hypothyroidism (SCH), defined as an elevated thyroid-stimulating hormone concentration with a normal free or total thyroxine concentration according to the study-specific reference limits. A normal triiodothyronine concentration was additionally required when it formed part of the source definition. An author-classified SCH category was accepted when the phenotype and integer count were explicit, even if a numerical TSH threshold was not reported. A universal cutoff was not imposed retrospectively because assays, participant ages, units, and reference intervals differed among studies. Accordingly, the primary synthesis pooled study-defined SCH phenotypes and should not be interpreted as representing a uniform biochemical threshold across all contributing studies. Each report was assigned to its final synthesis role only after its phenotype definitions, publication status, numerator, and denominator had been verified.

Overt or primary hypothyroidism, secondary or central hypothyroidism, isolated low-T3 or low-T4 patterns, euthyroid-sick or non-thyroidal-illness profiles, hyperthyroid profiles, and unclassified thyroid abnormalities were summarized descriptively. These outcomes were not combined as “any thyroid dysfunction” because they represent different, potentially overlapping pathophysiologic constructs and were often incompletely defined.

Two reviewers independently extracted data using a standardized form, resolving discrepancies by consensus. Extracted variables comprised publication year, country, study design and setting, CKD sample size, participant age, CKD stage or kidney-function classification, KRT status and modality, thyroid analytes, assay platform, reference intervals, diagnostic definitions, and phenotype-specific event counts. Additional study-level clinical variables included eGFR or measured GFR by CKD stage when available, proteinuria, serum albumin, nutritional or anthropometric measures, inflammatory markers, potentially thyroid-relevant medications or exposures, dialysis vintage and modality, and the timing of thyroid sampling relative to dialysis. The synthesis role of each report was also recorded. The CKD cohort, rather than any healthy control group, was the unit of analysis. Data collection was restricted to information available in the published reports. Contacting the original study authors to obtain additional laboratory information or individual participant data was not included in the review design and was not undertaken.

Each included full text and its relevant tables were rechecked before analysis. Published integer counts were prioritized. A percentage was converted to an integer only when the denominator and stratum-specific information supported a single defensible value; ambiguous rounded percentages were not imputed. Mutually exclusive categories were checked against the reported denominator, and overlapping percentages were not summed. If tables within a report conflicted, the internally coherent phenotype table was used for that outcome, the discrepancy was documented, and values were not averaged. Reconstructed numerators and denominators were verified against reported totals. These rules determined whether a report entered the primary meta-analysis, sensitivity analysis, or descriptive synthesis and were based on phenotype validity, publication status, and data recoverability, not on the magnitude or direction of its findings. Studies were not excluded from the systematic review solely because they could not contribute to a particular meta-analysis.

Generative artificial intelligence was not used for study design, literature selection, data extraction, risk-of-bias assessment, statistical analysis, interpretation, or manuscript drafting. ChatGPT (GPT-5.6 Sol; OpenAI, San Francisco, CA, USA) was used solely to assist with the graphical preparation of Figure 1 and Figure 2, which were reviewed and verified by the authors.

### 2.4. Risk-of-Bias Assessment Methods

Two reviewers independently appraised reports providing prevalence data using the nine-item JBI critical appraisal checklist for prevalence studies [18]. The checklist evaluates the sampling frame and recruitment, sample-size adequacy, description and coverage of the sample, validity and consistency of outcome measurement, appropriateness of the analysis, and response rate. Items were rated yes, no, unclear, or not applicable. No numerical summary score or exclusion threshold was used. Judgments informed interpretation but did not determine eligibility.

Outcome-reporting concerns were assessed by comparing the thyroid outcomes described in each report’s methods with the numerical data available in its text and tables. Particular attention was paid to missing assay information or diagnostic thresholds, participants already receiving thyroid treatment, and phenotype counts that could not be separated. These concerns were recorded as limitations of interpretation rather than converted into an additional numerical score.

### 2.5. Quantitative Synthesis and Statistical Analysis

The primary meta-analysis was restricted to peer-reviewed reports with a recoverable SCH numerator and CKD denominator that met the phenotype rules described above. The Ansari et al. preprint [22] was included only in a separate sensitivity analysis. Reports without a valid SCH numerator and denominator were retained for descriptive synthesis.

For each study, the observed SCH proportion and exact two-sided Clopper–Pearson 95% confidence interval (CI) were calculated. Proportions were transformed to the logit scale as y_i_ = log[x_i_/(n_i_ − x_i_)], with within-study sampling variance v_i_ = 1/x_i_ + 1/(n_i_ − x_i_), and pooled using an inverse-variance random-effects model. Study weights were calculated as the inverse of the sum of the within-study variance and estimated between-study variance. Between-study variance was estimated by restricted maximum likelihood (REML). The pooled 95% CI used a modified Hartung–Knapp method with a t distribution on k − 1 degrees of freedom and the residual variance scale constrained to a minimum of 1 [23,24]. This constraint prevented anticonservative narrowing when the estimated residual scale was below unity. No continuity correction was required because every study included at least one event and one non-event. Pooled estimates and confidence limits were back-transformed to the proportion scale.

When at least five studies were available, a 95% prediction interval was calculated on the logit scale using a t distribution with k − 2 degrees of freedom. Its standard error incorporated both the REML estimate of between-study variance and the modified Hartung–Knapp variance. Prediction limits were subsequently back-transformed to the proportion scale. Heterogeneity was quantified using Cochran’s Q, τ^2^, and I^2^. Given the small number of contributing studies, I^2^ was interpreted descriptively.

Robustness was evaluated using a binomial-normal random-intercept generalized linear mixed model fitted with 80-point Gauss–Hermite quadrature and a profile-likelihood 95% CI [25], leave-one-out reanalysis of the primary pool, and sensitivity analysis including the Ansari et al. preprint [22]. A post hoc exploratory sensitivity analysis was restricted to four studies that explicitly defined SCH using bounded TSH intervals together with normal thyroid hormone concentrations [10,11,12,13]. The reported intervals were 4.07–9.9 mU/mL [10], 5–10 mIU/mL [11], 4.07–9.9 μIU/mL [12], and 5–10 mIU/L [13]; units are reproduced as printed in the original publications. Study [19] defined SCH using TSH > 4.2 mU/L with normal free T4 but specified no upper TSH limit and therefore did not meet this narrower criterion; it remained included in the primary meta-analysis. Funnel plots and formal tests for small-study effects were not undertaken because fewer than 10 studies contributed to each quantitative analysis. All tests were two-sided with α = 0.05. Analyses were performed in Python 3.12.13 using double-precision routines from NumPy 2.3.5 and SciPy 1.17.0.

## 3. Results

### 3.1. Study Selection and Characteristics

The search identified 832 records. After removal of 243 duplicates, 589 records were screened, of which 542 were excluded. Of 47 reports sought for retrieval, three were not retrieved. The remaining 44 full-text reports were assessed for eligibility, 33 were excluded, and 11 studies met the eligibility criteria. The complete selection pathway and reasons for full-text exclusion are presented in Figure 1.

The 11 reports were published between 1977 and 2026 and included 694 children with CKD from seven countries. Cohort size ranged from 24 to 150 participants. The evidence covered CKD stages 1–5 and included conservatively managed children as well as selected cohorts receiving dialysis or transplantation. Seven of the 11 studies (63.6%), comprising 403 of 694 participants (58.1%), contributed to the primary peer-reviewed meta-analysis. One preprint (9.1%) was included only in the sensitivity analysis. Three reports (27.3%) were retained for descriptive or qualitative synthesis because a valid SCH numerator and denominator could not be recovered. The characteristics and synthesis roles of the included studies are summarized in Table 1.

Reporting of potential clinical modifiers of thyroid function varied substantially across studies. Detailed information on kidney-function severity, proteinuria, serum albumin, nutritional or anthropometric measures, inflammatory markers, relevant medications or exposures, and dialysis-related factors is provided in Supplementary Table S3. Several of these variables were incompletely reported, precluding meaningful study-level stratification.

### 3.2. Thyroid Phenotypes and Study-Level Findings

Definitions of SCH and the supporting laboratory methods varied across reports. Five of the seven primary studies (71.4%) reported a numerical TSH threshold together with normal thyroid hormone concentrations. Four specified a bounded TSH interval for SCH [10,11,12,13], whereas one specified a lower threshold without an upper limit [19]. The remaining two studies (28.6%) did not report a numerical TSH threshold [20,21]. Thus, although all seven studies provided recoverable study-defined SCH counts, the primary meta-analysis did not represent a completely standardized biochemical phenotype. Reported assay methods included radioimmunoassays and electrochemiluminescence platforms, although laboratory information was incomplete in several studies. Across the seven primary studies, SCH prevalence ranged from 6.1% [20] to 18.0% [10], with a crude aggregate of 50 cases among 403 children (12.4%). Study-specific definitions, laboratory information, SCH frequencies, and other thyroid findings are summarized in Table 2.

Other thyroid phenotypes were not pooled because their definitions and reported categories were not equivalent. One study identified isolated low T3 or low free T4 in 11 of 22 clinically euthyroid children and described two overt hypothyroid cases attributable to non-CKD causes [9]. Another study reported hypothyroidism in 8 of 51 children, but SCH could not be separated from other subtypes [26]. A further study reported elevated-TSH hypothyroidism in 25% of participants without providing a diagnostic threshold or a recoverable integer numerator [14]. These three reports were therefore retained only for qualitative or descriptive synthesis. In one of the primary studies [12], the reported total number of hypothyroidism cases differed by one participant between two tables, whereas the SCH numerator of seven was consistent.

The seven studies contributing to the primary meta-analysis reported no longitudinal thyroid-function outcomes sufficient to quantify persistence, normalization, or progression of SCH [10,11,12,13,19,20,21]. Two reports explicitly noted the absence of follow-up data [11,21].

### 3.3. Meta-Analysis of Subclinical Hypothyroidism

The primary random-effects meta-analysis of seven peer-reviewed studies yielded a pooled SCH prevalence of 12.8% (95% CI, 9.2–17.6%). The corresponding 95% prediction interval was 9.1–17.9%. No between-study heterogeneity was detected, with τ^2^ = 0.000, I^2^ = 0.0%, and Cochran’s Q = 4.19 (df = 6, *p* = 0.651) (Figure 2A and Table 3).

The binomial-normal GLMM produced a concordant pooled estimate of 12.4% (95% profile-likelihood CI, 9.3–15.9%). In the leave-one-out analyses, pooled estimates ranged from 12.0% after exclusion of Garrido-Magaña et al. [10] to 13.5% after exclusion of Santa et al. [21]. Both τ^2^ and I^2^ remained zero in every iteration, indicating that no individual peer-reviewed study materially altered the primary estimate.

The post hoc exploratory sensitivity analysis restricted to studies defining SCH using explicitly bounded TSH intervals together with normal thyroid hormone concentrations included four studies and 33 SCH cases among 225 children [10,11,12,13]. The pooled prevalence was 14.8% (95% CI, 8.7–24.1%), with no detected between-study heterogeneity (τ^2^ = 0.000, I^2^ = 0.0%, and Cochran’s Q = 0.97; df = 3; *p* = 0.808) (Table 3). The confidence interval was wider than in the primary analysis. This exploratory subset does not resolve uncertainty regarding the appropriateness of the lower TSH thresholds or individual case classifications.

When the Ansari et al. preprint [22] was added, the pooled SCH prevalence increased to 14.8% (95% CI, 9.3–22.7%). Heterogeneity increased to τ^2^ = 0.232 and I^2^ = 65.9%, with Cochran’s Q = 20.53 (df = 7, *p* = 0.005), while the 95% prediction interval widened to 4.5–38.8% (Figure 2B and Table 3). Inclusion of the preprint therefore increased the pooled estimate by 2.0 percentage points and substantially reduced between-study consistency.

### 3.4. Risk-of-Bias Assessment

Across the 11 included reports, the sampling frame, participant description, and coverage of the analyzed cohort were generally judged adequate. Recruitment procedures were insufficiently described in several reports, formal sample-size or precision justification was usually absent, and response-rate reporting was frequently incomplete. The principal concerns related to outcome measurement, as numerical diagnostic thresholds, age-specific reference intervals, and assay information were missing or incompletely reported in several studies. No report was excluded based on the risk-of-bias assessment. Detailed domain-level judgments are presented in Supplementary Table S2.

## 4. Discussion

This systematic review and meta-analysis provide a phenotype-specific estimate of subclinical hypothyroidism (SCH) in children with chronic kidney disease (CKD) is based on a deliberately narrower outcome definition than the broad and variably used label of thyroid dysfunction. In the primary analysis of seven peer-reviewed studies, SCH prevalence ranged from 6.1% to 18.0% at the study level, and the pooled prevalence was 12.8% (95% CI, 9.2–17.6%), with a 95% prediction interval of 9.1–17.9%. The corresponding binomial-normal generalized linear mixed model yielded a nearly identical estimate of 12.4%, and leave-one-out analyses remained between 12.0% and 13.5%. Thus, the most defensible interpretation is that approximately one in eight children in the included peer-reviewed CKD cohorts was classified as having SCH according to the study-specific definitions. Importantly, the seven studies contributing to this estimate represented different parts of the pediatric CKD spectrum, including dialysis-dependent children in the Mexican cohort, mixed CKD stages in Egypt, India, Iraq, and Bangladesh, and non-dialysis or pre-transplant populations [10,11,12,13,19,20,21].

The study-level pattern is clinically informative. Garrido-Magaña et al. reported SCH in 18.0% of a predominantly peritoneal-dialysis cohort, whereas Das et al. reported 16.0% in a pre-transplant population, and Yadav et al. reported 13.8% across CKD stages 1–5 [10,11,13]. Intermediate estimates were observed by Garbadi et al. and Mohamed et al. at approximately 12%, while lower frequencies were reported by Santa et al. and Obeed et al. [12,19,20,21]. Despite these clinical differences, no statistical heterogeneity was detected in the primary model. This numerical consistency is reassuring, but it should not be interpreted as proof that SCH prevalence is identical across CKD stages, kidney replacement therapy (KRT) modalities, geographic settings, or laboratory platforms. With only seven relatively small studies, heterogeneity statistics have limited power to detect real clinical variation, particularly when diagnostic thresholds and age-specific reference intervals are incompletely reported.

The sensitivity analysis including the preprint illustrates the influence of this additional report on the pooled estimate and between-study heterogeneity. The Ansari preprint reported SCH in 22 of 66 children (33.3%), considerably above the range of the peer-reviewed studies [22]. When this report was added, the pooled estimate increased from 12.8% to 14.8%, but the more important change was the emergence of substantial heterogeneity (I^2^ = 65.9%) and widening of the prediction interval to 4.5–38.8%. The preprint did not provide an operational TSH threshold, assay platform, or sufficiently detailed reference intervals to establish equivalence with the primary studies. Its higher estimate may therefore reflect a genuinely different population, a different diagnostic convention, or outcome misclassification. The present data cannot distinguish between these explanations. Including this report in a separate sensitivity analysis allowed its influence to be assessed while retaining the restriction of the primary analysis to peer-reviewed reports.

The distinction between SCH and other thyroid abnormalities is central to interpretation. The historical study by Wassner et al. found isolated low T3 or low free T4 in 11 of 22 clinically euthyroid children and described two overt hypothyroid cases that were attributed to non-CKD causes [9]. Hasan et al. reported hypothyroidism in 8 of 51 children, but the available report did not allow SCH to be separated reliably from other hypothyroid phenotypes [26]. Ohiri and Owamagbe reported elevated-TSH hypothyroidism in 25% of participants, but a numerical diagnostic threshold and an unambiguous integer numerator for SCH could not be recovered, and overlap with low free T3 or low free T4 categories was unclear [14]. These studies remain important components of the systematic review, but combining them quantitatively with studies reporting separately identifiable SCH counts would have conflated biologically different states and introduced avoidable classification error.

This phenotype-specific approach is also supported by the complex bidirectional physiology linking the kidney and thyroid. CKD can reduce peripheral conversion of thyroxine (T4) to triiodothyronine (T3), alter iodine clearance, modify thyroid-hormone binding and metabolism, disrupt hypothalamic–pituitary–thyroid signaling, and amplify the effects of chronic inflammation, malnutrition, metabolic acidosis, and intercurrent illness [3,4,5,27]. Classic and contemporary reviews have emphasized that these changes may produce low-T3 or non-thyroidal-illness patterns without primary thyroid failure and may also affect the interpretation of TSH and free hormone measurements [6,28,29]. In advanced CKD, impaired iodine excretion can increase the systemic iodine pool, while altered deiodinase activity and inflammatory signaling can suppress T3 concentrations. Consequently, a low T3 concentration, an elevated TSH concentration with preserved free T4, and overt hypothyroidism should not be regarded as interchangeable manifestations of one disorder.

The pediatric findings are broadly consistent with the adult CKD literature showing that hypothyroidism and SCH become more frequent as kidney function declines, although direct numerical comparisons are inappropriate because adult populations differ markedly in age, CKD etiology, comorbidity, and autoimmune risk. In a large population-based analysis, Lo et al. demonstrated a progressive increase in hypothyroidism prevalence with lower estimated glomerular filtration rate (eGFR), and Chonchol et al. similarly identified a substantial burden of SCH in adults with CKD [30,31]. A Japanese CKD cohort also linked hypothyroid phenotypes with lower eGFR, greater proteinuria, and lower serum albumin [32]. Proteinuria may be particularly relevant because urinary loss of thyroid hormones and hormone-binding proteins provides an additional mechanism by which renal disease can alter thyroid biochemistry. Adult CKD data have reported associations between hypothyroidism and proteinuria [33], but the pediatric evidence is not yet sufficiently standardized to determine whether proteinuria independently predicts persistent SCH.

The clinical meaning of a prevalence near 13% requires caution. Thyroid hormones are essential to normal somatic growth, pubertal maturation, neurodevelopment, metabolism, and cardiovascular function, and these domains are already vulnerable in children with CKD. Nevertheless, the present review estimates prevalence rather than consequences. Most included studies were cross-sectional and could not determine whether an elevated TSH was transient, persistent, or progressive. They also could not establish whether SCH independently contributes to growth failure, cardiovascular risk, neurocognitive outcomes, or CKD progression. Adult observational studies have associated thyroid status with kidney-function trajectories and have explored possible effects of levothyroxine therapy, but these data are vulnerable to confounding and cannot be used as direct evidence for pediatric treatment [34,35].

Protein loss and intercurrent renal disease further complicate attribution. In children with nephrotic syndrome, thyroid-hormone abnormalities may accompany heavy proteinuria and can improve when the nephrotic state resolves [36,37]. Although nephrotic syndrome is not equivalent to established CKD, these observations reinforce the principle that kidney-related thyroid abnormalities can be dynamic and mechanism-dependent. For a child with CKD and a newly elevated TSH concentration, interpretation should therefore consider current clinical stability, proteinuria, nutritional status, inflammatory burden, medication exposure, dialysis timing, iodine exposure, and the laboratory’s age-appropriate reference interval rather than assuming persistent primary thyroid dysfunction from a single measurement.

The importance of avoiding overinterpretation is heightened in pediatrics. Growth failure in CKD is multifactorial and reflects disturbances in nutrition, acid–base balance, mineral metabolism, growth hormone and insulin-like growth factor signaling, inflammation, and CKD severity [2,38]. Mild SCH in otherwise healthy children also has a heterogeneous natural history. The pediatric endocrine literature generally distinguishes mild SCH from more marked TSH elevation and recommends that management be individualized according to persistence, TSH magnitude, symptoms, goiter, autoimmunity, etiology, and associated conditions rather than based on a single biochemical value [7,8,39]. These principles are particularly relevant in CKD, where non-thyroidal influences on thyroid testing are common. The pooled prevalence reported here therefore supports clinical awareness and careful confirmation of the phenotype, but it does not by itself justify a CKD-specific universal treatment threshold.

Cardiovascular considerations are similarly important but remain insufficiently defined in children. SCH has been discussed as a potential contributor to dyslipidemia, vascular dysfunction, and cardiovascular risk in CKD, a population already characterized by markedly elevated cardiovascular burden [40]. However, most outcome data arise from adults. Likewise, observational adult studies reporting improvements in eGFR after thyroid-hormone replacement cannot establish that treating mild SCH prevents CKD progression, and they do not resolve the balance of benefit and overtreatment in children [41]. The relationship is also bidirectional because overt hypothyroidism itself can reduce renal blood flow and GFR and raise serum creatinine, changes that may reverse with correction of thyroid deficiency [42]. This reciprocal physiology makes causal inference from cross-sectional associations especially difficult.

The risk-of-bias findings and diagnostic heterogeneity limit the certainty and generalizability of the pooled estimate. Incomplete reporting of recruitment procedures and sample-size justification, together with incomplete reporting of diagnostic thresholds, age-specific reference intervals, and assay information, reduced confidence in phenotype classification. Importantly, the primary meta-analysis did not represent a completely standardized biochemical phenotype: four studies used explicitly bounded numerical TSH intervals [10,11,12,13], one used a lower numerical threshold without an upper limit [19], and two relied on qualitative or author-classified SCH definitions [20,21]. Because no additional information was sought from the original study authors, missing diagnostic thresholds and laboratory reference intervals could not be clarified beyond the published reports. Classification therefore relied on the original authors’ definitions and aggregate counts, with residual uncertainty regarding SCH classification. The post hoc exploratory analysis restricted to the four studies with explicitly bounded TSH intervals yielded a pooled prevalence of 14.8% (95% CI, 8.7–24.1%; I^2^ = 0.0%), compared with 12.8% (95% CI, 9.2–17.6%) in the primary analysis. The similar point estimates are reassuring, although the wider confidence interval and differences in thresholds, assays, and reference intervals preclude inference of diagnostic equivalence. In two studies, the lower TSH threshold for SCH was just above the stated upper normal limit (Table 2) [10,12]. Although these definitions were internally consistent with the reported reference intervals, the publications did not establish whether those intervals were manufacturer-derived or locally established for the assays and pediatric populations studied. Borderline values classified as SCH could therefore fall within the normal range if a higher upper reference limit were applied. Neither report provided sufficient data to determine how many SCH cases had borderline TSH values or to recalculate prevalence using a higher cutoff [10,12]. The pooled estimate should therefore be interpreted as reflecting study-defined SCH rather than a uniform biochemical phenotype. Potential clinical modifiers were also inconsistently reported across studies, particularly proteinuria, serum albumin, inflammatory markers, potentially thyroid-relevant medication use or iodine exposure, and dialysis-related sampling factors, limiting meaningful adjusted or stratified analyses. Across all included studies, publication years ranged from 1977 to 2026, and laboratory methods ranged from historical radioimmunoassays to contemporary electrochemiluminescence methods. These limitations reduce confidence in extrapolating a single prevalence estimate to every pediatric CKD setting.

Several statistical considerations also warrant emphasis. The primary analysis included only seven studies and 403 children. An I^2^ of 0% indicates that the observed dispersion was compatible with sampling error under the fitted model, not that the underlying populations, assays, or CKD phenotypes were equivalent. The narrow prediction interval in the primary analysis is encouraging, yet it is conditional on the limited set of studies currently available. Conversely, the sensitivity analysis demonstrates how rapidly between-study variability can increase when the preprint is added. The use of a random-effects framework with restricted maximum likelihood estimation, modified Hartung-Knapp inference, a binomial-normal generalized linear mixed model, and leave-one-out analyses was intended to make inference more robust in a small evidence base rather than to create precision that the underlying studies cannot support. The post hoc exploratory analysis restricted to four studies using explicitly bounded TSH intervals yielded a prevalence of 14.8%, with a wider confidence interval than the primary analysis. This result describes a narrower subset of published definitions and should not be interpreted as confirmation of diagnostic validity or equivalence across studies.

The review nevertheless has several strengths. It was conducted and reported within an explicit systematic-review framework and used methods developed for prevalence evidence [16,17]. Eligibility for the systematic review was separated from eligibility for a specific meta-analysis, allowing clinically informative studies to remain in the qualitative synthesis when their phenotype definitions or integer counts were not sufficiently recoverable for pooling. Outcome definitions and raw numerators were rechecked before synthesis, ambiguous rounded percentages were not imputed, and low-T3, overt hypothyroid, central hypothyroid, hyperthyroid, and unclassified patterns were not collapsed into a single composite endpoint. The statistical approach used restricted maximum likelihood for between-study variance, modified Hartung–Knapp inference to reduce anticonservative confidence intervals in a small meta-analysis, and a binomial-normal generalized linear mixed model as an independent robustness analysis [23,24,25]. Heterogeneity statistics were interpreted descriptively rather than as binary evidence of homogeneity, consistent with established cautions regarding I^2^ when few studies are available [43].

The pooled estimate describes SCH according to the study-specific definitions at the reported assessment; it does not establish the prevalence of persistent SCH. The absence of longitudinal thyroid-function outcomes in the contributing studies precluded assessment of spontaneous normalization or progression [10,11,12,13,19,20,21]. Repeat measurements in the same children, with documented testing intervals, clinical stability, and intervening thyroid treatment, are needed to distinguish transient from sustained abnormalities.

Future research should move beyond single-center cross-sectional prevalence studies. Prospective multicenter cohorts should use consecutive recruitment, prespecified sample-size calculations, age- and assay-specific reference intervals, and explicit definitions for SCH, overt hypothyroidism, low-T3 syndrome, and other non-thyroidal-illness patterns. Thyroid testing should be repeated during clinically stable periods to distinguish persistent SCH from transient abnormalities associated with acute illness, protein loss, dialysis timing, medication exposure, inflammation, malnutrition, or iodine imbalance. Reports should provide exact phenotype-specific numerators and denominators by CKD stage, eGFR category, proteinuria burden, and KRT modality. Longitudinal outcomes should include growth velocity, pubertal progression, neurocognitive measures, cardiovascular markers, kidney-function trajectory, health-related quality of life, and spontaneous normalization or progression of SCH. Only after a persistent and reproducible pediatric CKD phenotype has been established should interventional studies determine whether treatment improves patient-important outcomes rather than merely normalizing TSH.

## 5. Conclusions

In the available peer-reviewed pediatric CKD literature, approximately one in eight children was classified as having SCH according to study-specific definitions, with a pooled prevalence of 12.8% and consistent estimates across complementary statistical models and leave-one-out analyses. Sensitivity analyses highlight the importance of clear and standardized phenotype definitions and adequate reporting of diagnostic thresholds, reference intervals, and assay characteristics when estimating SCH prevalence. Low-T3 and other non-thyroidal-illness patterns should remain analytically and clinically distinct from SCH. Current evidence supports awareness of thyroid abnormalities, confirmation of persistent SCH using age-appropriate laboratory standards, and more rigorous longitudinal investigation. It does not yet define a pediatric CKD-specific screening interval, establish a universal treatment threshold for mild SCH, or demonstrate that routine levothyroxine therapy improves growth, cardiovascular, or kidney outcomes.

## Figures and Tables

**Figure 1:**
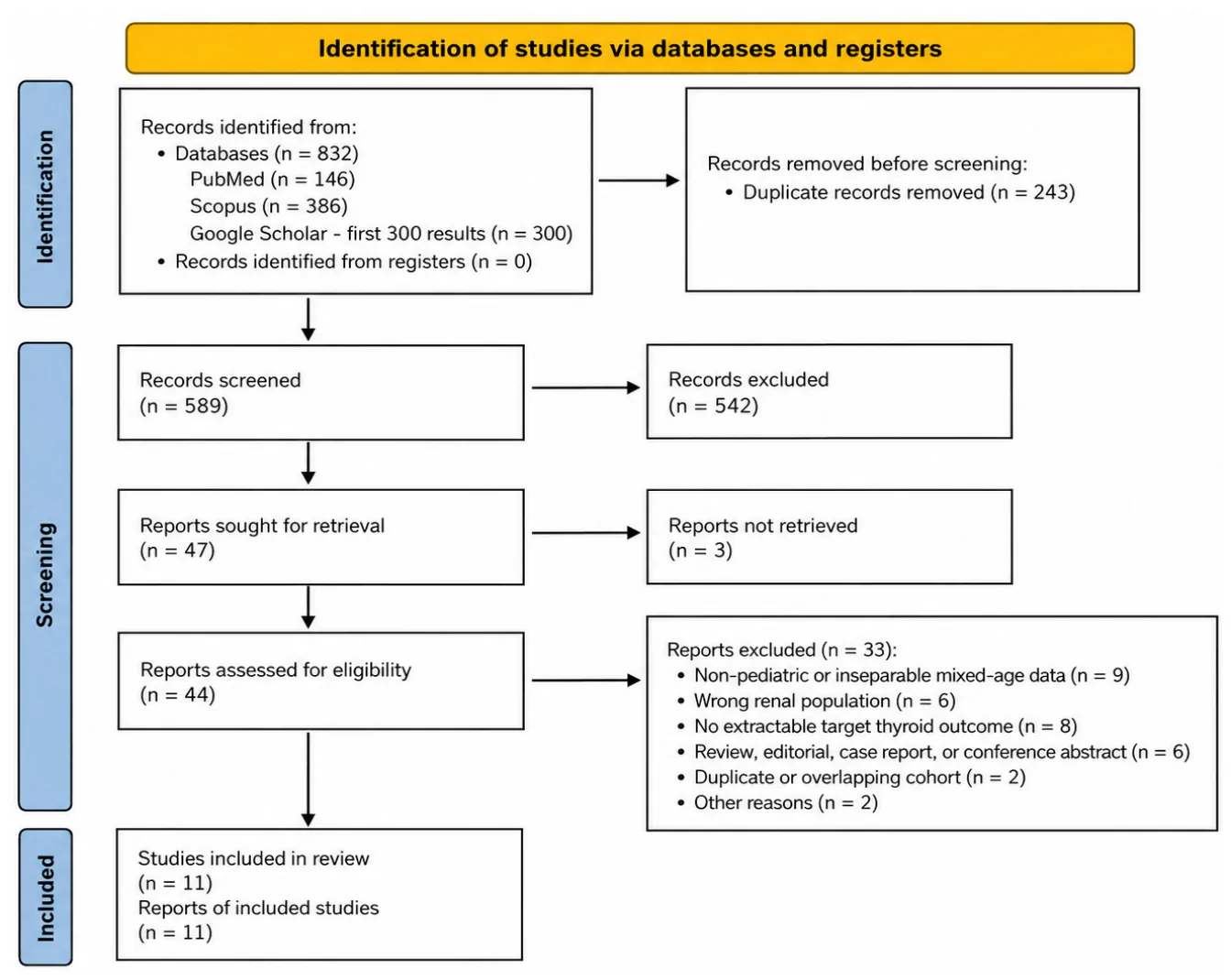
PRISMA 2020 flow diagram of the study identification and selection process.

**Figure 2:**
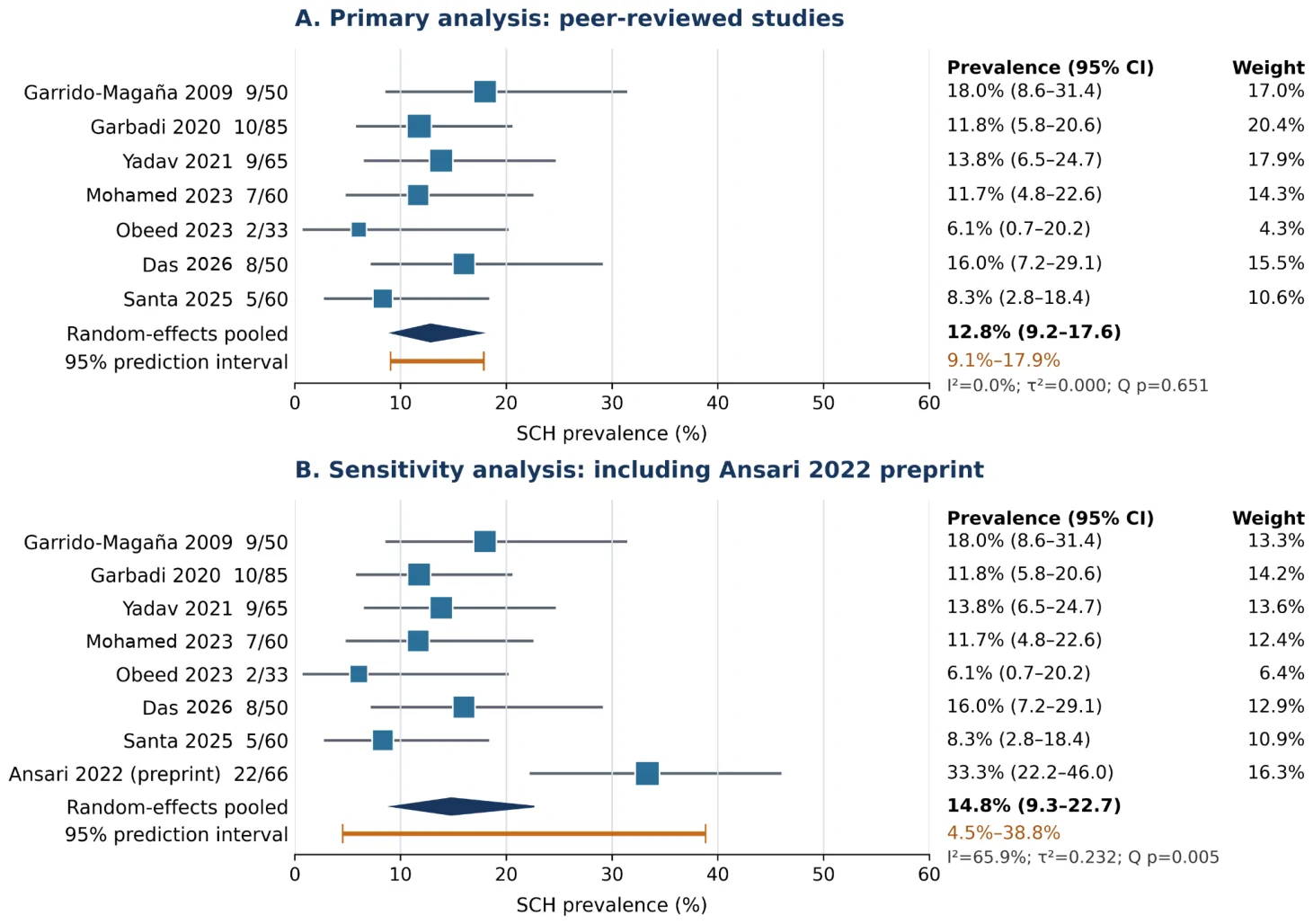
Random-effects meta-analysis of the prevalence of subclinical hypothyroidism in children with chronic kidney disease. (**A**) Primary analysis of seven peer-reviewed studies [10,11,12,13,19,20,21]. (**B**) Sensitivity analysis after inclusion of the Ansari et al. preprint [22]. Squares represent study-specific prevalence estimates, with their size proportional to the corresponding random-effects weight. Horizontal gray lines indicate exact two-sided Clopper–Pearson 95% confidence intervals (CIs). Diamonds represent the pooled prevalence estimates and their modified Hartung–Knapp 95% CIs. Orange horizontal lines indicate the 95% prediction intervals. Proportions were synthesized on the logit scale using an inverse-variance random-effects model, with between-study variance (τ^2^) estimated by restricted maximum likelihood. Statistical heterogeneity is reported using I^2^ and Cochran’s Q. CI, confidence interval; CKD, chronic kidney disease; REML, restricted maximum likelihood; SCH, subclinical hypothyroidism.

**Table 1:** Characteristics and synthesis roles of the 11 studies included in the systematic review.

Study	Country	Design and Setting	CKD Sample, *n*	Age, Years	CKD and KRT Characteristics	Synthesis Role
Garrido-Magaña 2009 [10]	Mexico	Cross-sectional descriptive study, tertiary pediatric center	50	4–17, mean 13	Chronic renal failure, PD *n* = 43, HD *n* = 7	Primary meta-analysis
Garbadi 2020 [19]	Egypt	Cross-sectional study with prospective recruitment, tertiary pediatric hospital	85	2–15, mean 7.65	Stages 1–5, conservative management *n* = 71, PD *n* = 4, HD *n* = 10	Primary meta-analysis
Yadav 2021 [11]	India	Cross-sectional study, tertiary teaching hospital	65	1–18, mean 7.86	Stages 1–5, conservative management only	Primary meta-analysis
Mohamed 2023 [12]	Egypt	Cross-sectional study with healthy controls, university center	60	2.9–16	Non-dialysis CKD, dialysis and transplantation excluded	Primary meta-analysis
Obeed 2023 [20]	Iraq	Case–control study, pediatric nephrology department	33	1.2–14, mean 7.18	Stages 2–5, conservative management only	Primary meta-analysis
Das 2026 [13]	India	Cross-sectional study, pediatric pre-transplant clinic	50	1–18, median 8	Stages 2–5, no KRT	Primary meta-analysis
Santa 2025 [21]	Bangladesh	Cross-sectional analytic study, tertiary pediatric nephrology center	60	2–18, mean 12.02	Stages 3–5, KRT status not reported	Primary meta-analysis
Ansari 2022 [22]	India	Prospective observational study, single center, preprint	66	<18, group means 6.8–7.0	Stages 3–5, KRT status not reported	Sensitivity analysis only
Wassner 1977 [9]	United States	Clinical and physiologic study	24	4–18	Chronic renal failure, predialysis *n* = 15, HD *n* = 9	Qualitative synthesis
Hasan 2025 [26]	Iraq	Cross-sectional study, two tertiary hospitals	51	1–17, mean 8.3	Stages 1–5, dialysis *n* = 7, kidney transplant *n* = 1	Descriptive synthesis
Ohiri 2025 [14]	Nigeria	Cross-sectional study, tertiary pediatric nephrology clinic	150	1 month–18 years, median 8 years	Stages 2–5, including KRT; stage 5/KRT combined *n* = 30	Descriptive synthesis

Age values are presented as ranges, with the mean or median added when reported. CKD terminology and stage classifications reflect the original reports and were not retrospectively reclassified. “Primary meta-analysis” denotes inclusion in the seven-study peer-reviewed analysis. The Ansari preprint [22] was included only in sensitivity analysis. CKD, chronic kidney disease; HD, hemodialysis; KRT, kidney replacement therapy; PD, peritoneal dialysis.

**Table 2:** Definitions and Study-Level Findings for Subclinical Hypothyroidism and Other Thyroid Phenotypes.

Study	SCH Definition and Laboratory Information	SCH Cases/Total (%)	Other Thyroid Findings and Review Notes
Garrido-Magaña 2009 [10]	TSH 4.07–9.9 mU/mL with normal T3 and free T4; stated normal TSH range, 0.17–4.06 mU/mL. T3/T4 measured by RIA and TSH by IRMA (Immunotech Beckman Coulter). *	9/50 (18.0)	Primary hypothyroidism, 2/50. ESS, 3/50.
Garbadi 2020 [19]	Elevated TSH (>4.2 mU/L) with normal free T4. Reported reference intervals: TSH, 0.4–4.2 mU/L; free T4, 0.93–1.7 ng/dL. No upper TSH limit for SCH was specified. Assay platform not reported.	10/85 (11.8)	Hypothyroidism, 2/85. Isolated low T3, 18/85.
Yadav 2021 [11]	TSH 5–10 mIU/mL with normal free T3 and free T4. ECL using a Cobas analyzer.	9/65 (13.8)	Overt hypothyroidism, 3/65. Isolated low T3, 5/65.
Mohamed 2023 [12]	TSH 4.07–9.9 μIU/mL with normal thyroid hormone concentrations; stated normal TSH range, 0.5–4.06 μIU/mL. BT-3500 analyzer; TSH assay principle and reagent details not reported. *	7/60 (11.7)	Primary hypothyroidism, 2/60. Secondary hypothyroidism, 2/60. NTI, 6/60. The SCH count was internally consistent, although the reported total hypothyroidism count differed between tables.
Obeed 2023 [20]	Author-classified SCH without an operational threshold. ECL using a Cobas e 411 analyzer.	2/33 (6.1)	Isolated low T3, 16/33. Isolated low T4, 3/33. Combined low T3/low T4, 5/33. Subclinical hyperthyroidism, 1/33.
Das 2026 [13]	TSH 5–10 mIU/L with normal free T4. ECL was reported, although platform information was not fully consistent.	8/50 (16.0)	Overt hypothyroidism, 5/50.
Santa 2025 [21]	Elevated TSH with normal thyroid hormone concentrations. Numerical threshold and assay platform not reported.	5/60 (8.3)	Isolated low T3, 35/60. Hyperthyroidism, 1/60. An additional “other” category included 10/60 participants but was not defined.
Ansari 2022 [22]	SCH reported without an operational threshold or assay information. Preprint included only in sensitivity analysis.	22/66 (33.3)	Hypothyroidism, 6/66, without sufficient information to verify subtype classification.
Wassner 1977 [9]	No explicit SCH definition. Historical radioimmunoassays were used, and normal TSH was reported as <10 μU/mL.	NR	Two overt hypothyroid cases were attributed to non-CKD causes. Isolated low T3 or low free T4 occurred in 11/22 clinically euthyroid participants. Not included in quantitative synthesis.
Hasan 2025 [26]	Elevated TSH with normal free T4. Assay platform was not reported, and age-specific ranges were derived from an external source.	NR	Hypothyroidism was reported in 8/51 participants, but SCH could not be separated from other subtypes. Not included in quantitative synthesis.
Ohiri 2025 [14]	No operational SCH definition or numerical TSH threshold. The assay platform was not reported.	NR	Elevated-TSH hypothyroidism was reported in 25%, but a valid integer numerator could not be recovered. Low free T4 occurred in 15% and low free T3 in 10%, with unknown overlap. Not included in quantitative synthesis.

The seven peer-reviewed studies [10,11,12,13,19,20,21] constituted the primary meta-analysis and included 50 SCH cases among 403 children. The Ansari et al. preprint [22] was added only in sensitivity analysis. SCH frequency is shown only when a valid numerator and denominator could be recovered. ECL, electrochemiluminescence; ESS, euthyroid sick syndrome; IRMA, immunoradiometric assay; NR, not recoverable; NTI, non-thyroidal illness; RIA, radioimmunoassay; SCH, subclinical hypothyroidism; TSH, thyroid-stimulating hormone. * In these studies [10,12], the SCH thresholds exceeded the stated upper normal TSH limits. However, the published information was insufficient to confirm whether these reference intervals were manufacturer-derived or locally established for the assays and pediatric populations studied. TSH units are reproduced as reported in the original publications.

**Table 3:** Summary of Quantitative Analyses.

Analysis	k	Events/N	Pooled Prevalence (95% CI)	95% PI	Heterogeneity
Primary: logit REML with modified Hartung–Knapp inference	7	50/403	12.8% (9.2–17.6%)	9.1–17.9%	τ^2^ = 0.000, I^2^ = 0.0%
Primary: binomial-normal GLMM	7	50/403	12.4% (9.3–15.9%)	—	τ = 0.0003
Sensitivity: inclusion of the Ansari 2022 preprint [22]	8	72/469	14.8% (9.3–22.7%)	4.5–38.8%	τ^2^ = 0.232, I^2^ = 65.9%
Post hoc exploratory sensitivity analysis: studies defining SCH using explicitly bounded TSH intervals [10,11,12,13]	4	33/225	14.8% (8.7–24.1%)	—	τ^2^ = 0.000, I^2^ = 0.0%

The primary and sensitivity estimates were obtained using a logit-transformed REML random-effects model with modified Hartung–Knapp inference. The binomial-normal GLMM was fitted as a random-intercept model, and its 95% CI was obtained by profile likelihood. A prediction interval and I^2^ were not estimated for the GLMM. The exploratory subset comprised studies specifying both lower and upper TSH limits for SCH, together with normal thyroid hormone concentrations [10,11,12,13]. This restriction was defined post hoc and does not establish the equivalence or validity of the study-specific thresholds. CI, confidence interval; GLMM, generalized linear mixed model; PI, prediction interval; REML, restricted maximum likelihood; SCH, subclinical hypothyroidism; TSH, thyroid-stimulating hormone; k, number of studies.

## Data Availability

All data supporting the findings of this study were derived from the published studies included in the systematic review and are presented within the article and its Supplementary Materials. No new primary datasets were generated.

## Supplementary Materials

The following supporting information can be downloaded at https://www.mdpi.com/article/10.3390/medsci14060607/s1: Table S1: Full electronic search strategies for all databases; Table S2: Detailed study-level JBI critical appraisal [9,10,11,12,13,14,19,20,21,22,26]; Table S3: Study-level reporting of CKD severity and potential clinical modifiers of thyroid function across included studies [9,10,11,12,13,14,19,20,21,22,26]; File S1: PRISMA 2020 checklist 16.

## Abbreviations

The following abbreviations are used in this manuscript:

CI	Confidence interval
CKD	Chronic kidney disease
ECL	Electrochemiluminescence
eGFR	Estimated glomerular filtration rate
ESS	Euthyroid sick syndrome
GFR	Glomerular filtration rate
GLMM	Generalized linear mixed model
HD	Hemodialysis
IRMA	Immunoradiometric assay
JBI	Joanna Briggs Institute
KRT	Kidney replacement therapy
NR	Not recoverable
NTI	Non-thyroidal illness
PD	Peritoneal dialysis
PI	Prediction interval
PRISMA	Preferred Reporting Items for Systematic Reviews and Meta-Analyses
PROSPERO	International Prospective Register of Systematic Reviews
RIA	Radioimmunoassay
REML	Restricted maximum likelihood
SCH	Subclinical hypothyroidism
T3	Triiodothyronine
T4	Thyroxine
TSH	Thyroid-stimulating hormone
