# Supplementary material for: Thyroid Dysfunction in Children with Chronic Kidney Disease: A Systematic Review and Meta-Analysis
Source: Med Sci (Basel). 2026 Sep 27;14(6):607. doi: 10.3390/medsci14060607 (PMC13706913; doi:10.3390/medsci14060607)
Supplement: Supplementary file 1 [file medsci-14-00607-s001.zip › Table_S1_Full_Electronic_Search_Strategies(8).pdf]

Table S1. Full electronic search strategies for all databases

Source / platform	Search date and coverage	Full search strategy	Records
PubMed/MEDLINE	31 March 2026<br>Database inception to search date	("Thyroid Diseases"[Mesh] OR "Hypothyroidism"[Mesh] OR "Thyroid Hormones"[Mesh] OR "Thyrotropin"[Mesh] OR "thyroid dysfunction"[Title/Abstract] OR hypothyroid*[Title/Abstract] OR "subclinical hypothyroidism"[Title/Abstract] OR thyroid hormone*[Title/Abstract] OR TSH[Title/Abstract] OR thyrotropin[Title/Abstract] OR T3[Title/Abstract] OR triiodothyronine[Title/Abstract] OR T4[Title/Abstract] OR thyroxine[Title/Abstract]) AND ("Renal Insufficiency, Chronic"[Mesh] OR "Kidney Failure, Chronic"[Mesh] OR "chronic kidney disease"[Title/Abstract] OR CKD[Title/Abstract] OR "chronic renal insufficiency"[Title/Abstract] OR "chronic renal failure"[Title/Abstract] OR "kidney failure"[Title/Abstract]) AND ("Child"[Mesh] OR "Adolescent"[Mesh] OR "Infant"[Mesh] OR child*[Title/Abstract] OR adolescen*[Title/Abstract] OR infant*[Title/Abstract] OR pediatric*[Title/Abstract] OR paediatric*[Title/Abstract])<br><br>Filters/limits: none.	146
Scopus	31 March 2026<br>Database inception to search date	TITLE-ABS-KEY("thyroid dysfunction" OR hypothyroid* OR "subclinical hypothyroidism" OR "thyroid hormone*" OR TSH OR thyrotropin OR T3 OR triiodothyronine OR T4 OR thyroxine) AND TITLE-ABS-KEY("chronic kidney disease" OR CKD OR "chronic renal insufficiency" OR "chronic renal failure" OR "kidney failure") AND TITLE-ABS-KEY(child* OR adolescen* OR infant* OR pediatric* OR paediatric*)<br><br>Filters/limits: none.	386
Google Scholar	31 March 2026<br>Database inception to search date	("thyroid dysfunction" OR hypothyroid* OR "subclinical hypothyroidism" OR "thyroid hormone*" OR TSH OR thyrotropin OR T3 OR triiodothyronine OR T4 OR thyroxine) AND ("chronic kidney disease" OR CKD OR "chronic renal insufficiency" OR "chronic renal failure" OR "kidney failure") AND (child* OR adolescen* OR infant* OR pediatric* OR paediatric*)<br><br>Limits: the first 300 results ranked by relevance were screened.	300

Additional searching: Reference lists of eligible reports and relevant reviews were examined. Targeted supplementary searching was completed on 1 August 2026. This was an additional, non-prespecified check before submission, not prompted by peer review, and involved targeted searches rather than a full repetition of the original Boolean strategies across all three sources. No additional eligible studies were identified through reference-list screening or targeted supplementary searching.
