# Supplementary material for: Thyroid Dysfunction in Children with Chronic Kidney Disease: A Systematic Review and Meta-Analysis
Source: Med Sci (Basel). 2026 Sep 27;14(6):607. doi: 10.3390/medsci14060607 (PMC13706913; doi:10.3390/medsci14060607)
Supplement: Supplementary file 1 [file medsci-14-00607-s001.zip › Table_S2_JBI_Critical_Appraisal(8).pdf]

Table S2. Detailed study-level JBI critical appraisal

Judgment codes: Y, yes; N, no; U, unclear; NA, not applicable. Judgments reflect information reported in each publication; U was used when reporting was insufficient. No numerical summary score or exclusion threshold was applied.

Study	Q1	Q2	Q3	Q4	Q5	Q6	Q7	Q8	Q9	Study-specific basis for concern
**Garrido-Magaña 2009 [10]**	Y	U	U	Y	Y	Y	Y	Y	U	Diagnostic threshold and laboratory methods were explicit; recruitment and response-rate reporting were incomplete.
**Garbadi 2020 [19]**	Y	Y	U	Y	Y	U	U	Y	Y	Prospective recruitment was reported; SCH was defined as TSH >4.2 mU/L with normal free T4. Laboratory reference intervals were provided, but assay methods and platform were insufficiently described to establish diagnostic validity and measurement reliability.
**Yadav 2021 [11]**	Y	Y	U	Y	Y	Y	Y	Y	Y	Numerical SCH definition and ECL platform were reported; no formal prevalence precision justification was identified.
**Mohamed 2023 [12]**	Y	U	U	Y	Y	U	U	U	U	Analyzer and threshold were reported, but assay detail was incomplete and hypothyroidism totals differed between tables; recruitment/response reporting was incomplete.
**Obeed 2023 [20]**	Y	U	U	Y	Y	N	Y	Y	U	SCH was author-classified without an operational threshold; the analyzer was reported, but recruitment and response-rate details were incomplete.
**Das 2026 [13]**	Y	Y	Y	Y	Y	U	U	Y	Y	A numerical SCH definition was reported, but assay-platform information was not fully consistent.
**Santa 2025 [21]**	Y	U	U	Y	Y	N	U	Y	U	Numerical threshold and assay platform were not reported, and an additional thyroid category was undefined.
**Ansari 2022 [22]**	Y	Y	U	Y	Y	N	U	Y	Y	Preprint with no operational SCH threshold or assay information; retained only for sensitivity analysis.
**Wassner 1977 [9]**	Y	U	U	Y	Y	U	Y	Y	U	Recruitment and response rate were not fully described; historical methods were reported, but no explicit SCH definition was provided.
**Hasan 2025 [26]**	Y	U	U	Y	Y	N	U	Y	U	Assay platform was not reported, external reference ranges were used, and SCH could not be separated from other hypothyroidism subtypes.
**Ohiri 2025 [14]**	Y	Y	U	Y	Y	N	U	U	U	No operational SCH threshold or assay platform; only rounded percentages were reported and an integer numerator was not recoverable.

JBI appraisal domains

Domain	JBI checklist question
**Q1**	Was the sample frame appropriate to address the target population?
**Q2**	Were study participants sampled in an appropriate way?
**Q3**	Was the sample size adequate?
**Q4**	Were the study subjects and setting described in detail?
**Q5**	Was the data analysis conducted with sufficient coverage of the identified sample?
**Q6**	Were valid methods used for identification of the condition?
**Q7**	Was the condition measured in a standard, reliable way for all participants?
**Q8**	Was there appropriate statistical analysis?
**Q9**	Was the response rate adequate, and if not, was the low response rate managed appropriately?
