# Supplementary material for: Thyroid Dysfunction in Children with Chronic Kidney Disease: A Systematic Review and Meta-Analysis
Source: Med Sci (Basel). 2026 Sep 27;14(6):607. doi: 10.3390/medsci14060607 (PMC13706913; doi:10.3390/medsci14060607)
Supplement: Supplementary file 1 [file medsci-14-00607-s001.zip › Table_S3_Thyroid_CKD(3).pdf]

Table S3. Study-level reporting of CKD severity and potential clinical modifiers of thyroid function across included studies

Study	Kidney function / CKD severity	Proteinuria	Serum albumin	Nutritional status / anthropometry	Inflammatory markers (CRP/IL-6)	Potentially thyroid-relevant medications / exposures	Dialysis / KRT modality and vintage	Timing of thyroid sampling relative to dialysis
**Garrido-Magaña 2009 [10]**	Chronic renal failure; numerical eGFR/mGFR NR.	NR	NR	Weight-for-age, height-for-age, and weight-for-height Z scores assessed. Height-for-age was impaired in 66%; weight-for-height was normal in all participants.	NR	NR	PD 43/50; HD 7/50. All had received dialysis for >3 months; median duration of kidney replacement therapy at evaluation, 1 year.	For HD participants, thyroid blood sampling was performed before dialysis.
**Garbadi 2020 [19]**	CKD stages 1–5. Mean eGFR 52.64 ± 27.28 (range 7.50–95.50) mL/min/1.73 m². Stage 1: 4/85; stage 2: 33/85; stage 3: 25/85; stage 4: 11/85; stage 5: 12/85.	NR	NR	Short stature reported in 33/85 (38.8%). Mean BMI 16.65 ± 4.20 kg/m²; underweight 20/85 (23.5%), normal weight 57/85 (67.1%), overweight 5/85 (5.9%), obesity 3/85 (3.5%).	NR	Reviewer-specified thyroid-relevant medication/exposure use was NR. General discussion of possible drug effects was not treated as cohort exposure.	Conservative management 71/85; PD 4/85; HD 10/85. Reported dialysis duration: PD 3.8 ± 1.3 months; HD 36.2 ± 26 months.	NR
**Yadav 2021 [11]**	CKD stages 1–5. Stages 1–3: n=43, GFR 59.7 ± 25.8; stages 4–5: n=22, GFR 18.4 ± 8.2 (units reported by the study). No participant was receiving dialysis.	NR	NR	Height SDS: −0.853 ± 1.42 (stages 1–3) vs −2.02 ± 1.67 (stages 4–5). Weight SDS: 0.274 ± 1.24 vs 0.129 ± 1.28, respectively.	NR	All participants were receiving iron, calcium carbonate, active vitamin D analogues, and sodium bicarbonate. The indication for calcium carbonate was not separately specified.	No dialysis; N/A.	N/A
**Mohamed 2023 [12]**	Non-dialysis CKD. eGFR categories: ≥90, n=8; 60–89, n=6; 45–59, n=2; 30–44, n=1; <30, n=43.	24-h urinary protein excretion and urinary albumin/creatinine ratio were measured; numerical results NR. Patients with heavy proteinuria >3000 mg/day were excluded.	NR	Weight, height, Tanner stage, and weight-for-age, height-for-age, and weight-for-height Z scores were assessed.	NR	Use of lithium or amiodarone was excluded. History of iodine-containing drugs, iodinated contrast exposure, or iodine-containing nuclear studies was also excluded.	Hemodialysis and peritoneal dialysis were exclusion criteria; N/A.	N/A

Study	Kidney function / CKD severity	Proteinuria	Serum albumin	Nutritional status / anthropometry	Inflammatory markers (CRP/IL-6)	Potentially thyroid-relevant medications / exposures	Dialysis / KRT modality and vintage	Timing of thyroid sampling relative to dialysis
**Obeed 2023 [20]**	CKD stages 2–5; eGFR calculated using the updated Schwartz equation. Stage 2: 4/33; stage 3: 11/33; stage 4: 13/33; stage 5: 5/33. Continuous stage-specific eGFR values NR.	General urinalysis reported; quantitative proteinuria NR.	Serum albumin NR; total serum protein was measured.	Nutritional status was recorded, but quantitative anthropometric/nutritional results were NR.	NR	Patients with a history of medication affecting thyroid function, including steroid therapy, were excluded.	Peritoneal dialysis and hemodialysis were exclusion criteria; N/A.	N/A
**Das 2026 [13]**	CKD stages 2–5; stage 2/3, n=13; stage 4/5, n=37. Median eGFR 16.0 (IQR 8.8–34.4) mL/min/1.73 m².	Spot UP:UC median 1.7 (IQR 0.8–3.8). UP:UC <0.2: 7/50; 0.2–2: 20/50; >2: 23/50.	NR	Median HAZ −2.2 (IQR −3.7 to −1.4); WAZ −2.25 (−2.8 to −1.4); BMIZ −1.3 (−1.9 to −0.2).	CRP/IL-6 NR	Reviewer-specified thyroid-relevant medications/exposures NR.	None of the participants were receiving kidney replacement therapy; N/A.	N/A
**Santa 2025 [21]**	CKD stage III, n=9; stage IV, n=11; stage V, n=40. CKD staging was based on eGFR; continuous numerical eGFR values NR.	NR	NR	NR	CRP/IL-6 NR	Patients receiving thyroid-replacement or antithyroid medication were excluded. Other reviewer-specified medications/exposures NR.	KRT status, modality, and vintage NR.	NR
**Ansari 2022 [22]**	CKD stages 3–5. Mean eGFR by thyroid category: SCH 21.14 ± 12.19; overt hypothyroidism 22.08 ± 21.05; euthyroid 21.74 ± 12.46 mL/min/1.73 m².	Proteinuric CKD: 4/22 with SCH, 1/6 with overt hypothyroidism, and 10/38 euthyroid.	Serum albumin was measured; numerical results NR.	Height and weight reported by thyroid phenotype: SCH 105.95 ± 15.19 cm and 16.41 ± 6.27 kg; overt hypothyroidism 102.17 ± 24.01 cm and 15.02 ± 6.29 kg; euthyroid 105.47 ± 16.28 cm and 16.58 ± 6.59 kg.	CRP/IL-6 NR	Iron: 21/22 SCH, 5/6 overt hypothyroidism, 36/38 euthyroid. Calcium supplementation: 22/22, 6/6, and 38/38, respectively. The report did not establish that calcium supplementation represented a calcium-based phosphate binder.	KRT status, modality, and vintage NR.	NR

Study	Kidney function / CKD severity	Proteinuria	Serum albumin	Nutritional status / anthropometry	Inflammatory markers (CRP/IL-6)	Potentially thyroid-relevant medications / exposures	Dialysis / KRT modality and vintage	Timing of thyroid sampling relative to dialysis
**Wassner 1977 [9]**	Chronic renal failure; 15 studied before first HD and 9 after >3 months of regular HD. Numerical eGFR/mGFR NR.	NR	Among clinically euthyroid participants, serum albumin was 3.4 ± 0.2 g/100 mL in the predialysis group and 3.7 ± 0.2 g/100 mL in the maintenance-HD group.	Bone age was >2 SD below chronological age in 11/21 clinically euthyroid children; cohort-level height/weight/BMI data NR.	NR	Current medications and recent radiographic contrast or nuclear-medicine studies were reviewed, but cohort-level exposure results were NR.	Predialysis n=15; maintenance HD n=9 after >3 months of dialysis.	In HD participants, blood was obtained at the start of dialysis, before heparin administration.
**Hasan 2025 [26]**	CKD stages 1–5: stage 1, n=7; stage 2, n=8; stage 3, n=10; stage 4, n=14; stage 5, n=12. Overall eGFR 44.11 ± 42.75 mL/min/1.73 m²; stage-specific eGFR values NR.	NR	NR	Weight 26 ± 21 kg; height 113 ± 24 cm; BMI 18.67 ± 9.14 kg/m². Underweight by weight class 4/51; short stature 27/51; BMI underweight 7/51, overweight 3/51, obese 6/51.	CRP/IL-6 NR	Concurrent medications were recorded but reviewer-specified thyroid-relevant exposures were not systematically reported. Thyroxine use was reported in 8/51.	Dialysis 7/51; kidney transplant 1/51. Dialysis modality and dialysis vintage NR. CKD duration (38 ± 29 months) should not be interpreted as dialysis vintage.	NR
**Ohiri 2025 [14]**	CKD stage 2, n=30; stage 3, n=50; stage 4, n=40; stage 5/RRT combined, n=30. eGFR was calculated using the Schwartz equation; continuous/stage-specific numerical eGFR values NR.	NR	NR	Weight, height, and BMI were collected to assess growth; numerical anthropometric results NR.	CRP/IL-6 NR	Patients with prior thyroid disease or taking thyroid medications were excluded. Other reviewer-specified thyroid-relevant medications/exposures NR.	RRT was represented within the combined stage 5/RRT group; modality and vintage NR.	Blood samples were collected in the morning; relation to a dialysis session was NR.

Notes: NR, not reported or not numerically recoverable from the published report; N/A, not applicable; BMIZ, body mass index-for-age Z score; CKD, chronic kidney disease; CRP, C-reactive protein; eGFR, estimated glomerular filtration rate; HAZ, height-for-age Z score; HD, hemodialysis; IL-6, interleukin-6; IQR, interquartile range; KRT, kidney replacement therapy; mGFR, measured glomerular filtration rate; PD, peritoneal dialysis; RRT, renal replacement therapy; SCH, subclinical hypothyroidism; UP:UC, urine protein-to-creatinine ratio; WAZ, weight-for-age Z score. NR should not be interpreted as evidence that the variable or exposure was absent. Where a variable was described as measured but numerical results were unavailable, this is stated explicitly. Only medications or exposures explicitly documented in the original report are shown. CKD stage and treatment terminology are retained as reported in the original studies.
